# The contribution of the basal ganglia and cerebellum to motor learning: A neuro-computational approach

**DOI:** 10.1371/journal.pcbi.1011024

**Published:** 2023-04-03

**Authors:** Javier Baladron, Julien Vitay, Torsten Fietzek, Fred H. Hamker

**Affiliations:** 1 Department of Computer Science, Chemnitz University of Technology, Chemnitz, Germany; 2 Departamento de Ingeniería Informática, Universidad de Santiago de Chile, Santiago, Chile; Ben-Gurion University, ISRAEL

## Abstract

Motor learning involves a widespread brain network including the basal ganglia, cerebellum, motor cortex, and brainstem. Despite its importance, little is known about how this network learns motor tasks and which role different parts of this network take. We designed a systems-level computational model of motor learning, including a cortex-basal ganglia motor loop and the cerebellum that both determine the response of central pattern generators in the brainstem. First, we demonstrate its ability to learn arm movements toward different motor goals. Second, we test the model in a motor adaptation task with cognitive control, where the model replicates human data. We conclude that the cortex-basal ganglia loop learns via a novelty-based motor prediction error to determine concrete actions given a desired outcome, and that the cerebellum minimizes the remaining aiming error.

## Author summary

In this study, we aimed to better understand motor learning using a neuro-computational approach. While previous work emphasized different learning regimes of the various components, the main novelty of our study is the interplay of its components. Notably, we show that the model accounts well for motor adaptation data of experiments that involve a cognitive strategy.

## Introduction

A commonly assumed role for the motor basal ganglia (BG) is action or motor program selection [1–6]. The basal ganglia integrate sensory evidence arguing for a particular decision and disinhibit the corresponding action plan. Such motor program selection involves a focal removal of tonic neural activity in the output nuclei of the BG to activate the desired movement while increasing other neuronal activity to avoid the execution of unwanted programs [7, 8]. However, how proper actions are discovered and represented is still unclear.

Although most common tasks addressed by computational models of the basal ganglia only require choosing a correct action among other actions, e.g. selecting a button as a response to sensory input [9–13], some models addressed the role of the BG in a broader context of the motor system. Magdoom et al. [14] proposed a computational model where the BG makes corrections to movements controlled by the motor cortex. The model was used to show how reduced dopamine signals in Parkinson’s disease can produce abnormal movements. In an extended version of this model, the BG amplified low amplitude input signals through stochastic resonance to produce movements [15]. Kim et al. [16] proposed a model where the BG selects a muscle activation pattern demonstrated by a 2-dimensional reaching task. In the motor control framework proposed by Manella and Baldasarre [17], the BG modulates the dynamics of a cortical reservoir that implements a movement. The authors reproduce three different periodic behaviors of a two-joint arm.

The cerebellum is crucial for maintaining accuracy across multiple movements [18, 19]. Individuals with cerebellar damage have deficits when required to adapt a well-known behavior to a sudden change in environmental conditions [20]. Its role in motor adaptation has been further confirmed in imaging studies [21, 22]. Cerebellar pathology has also been hypothesized to be related to the Developmental Coordination Disorder, which in children manifests as a reduced motor performance [23, 24]. The cerebellum may implement a forward model, an inverse model, or both. Like with the basal ganglia, most models of the cerebellum focus on internal dynamics but are rarely applied to complex motor tasks. Those computational models of the cerebellum involved in motor tasks have been mainly developed in the context of neuro-robotics, often abstract much from biological detail and typically implement a closed-loop motor control network [25–31].

Influential theories regarding the interaction between different motor systems emphasize that each system operates with a different type of learning mechanism, with the cerebellum implementing supervised learning, the basal ganglia reinforcement learning, and the cortex unsupervised learning [32]. An extended version of this idea, the super-learning hypothesis, proposes that the three learning mechanisms form an integrated system and act in synergy [33]. Results from one system may influence another through multiple neural pathways or neuromodulators.

Houk and colleagues [34, 35] proposed a conceptual framework that suggests distributed processing modules. It places the cerebral cortex at the center, and independent loops with the BG and cerebellum feed back to the cortex. The cortico-basal ganglia loops make an initial course selection that is then narrowed down (or refined) by the corresponding cortico-cerebellar loop. For example, in a reaching task, the cerebellum may use a prediction error to compute an online corrective movement. Thus, although the cerebellum, cortex, and basal ganglia may use different learning paradigms, they implement an interactive system capable of handling a diversity of tasks [36–38].

Shadmehr and Krakauer [39] proposed a theory based on the framework of optimal feedback control. Rather than action selection, the basal ganglia are given a higher-level involvement in planning with respect to the cost and reward structure of the task. The cerebellum implements a forward model to predict the sensory consequences of motor action [40]. Recently, Haar and Donchin [41] combined Houk’s approach [34] with Shadmehr and Krakauer’s optimal control theory. They emphasize the distributed nature of the cortical network. The cortex-cerebellum loops are assumed to implement a predictive error correction of the cortical activity. However, their theory assumes the concept of parallel and segregated cortex-basal ganglia loops and thus, underestimates recent evidence for a hierarchical organization of cortex-basal ganglia loops [37, 42–44].

In addition to the rather theoretical frameworks discussed above, the interaction of brain areas relevant to motor tasks can be explored by means of computational models. Very few computational models have included both, the cerebellum and the basal ganglia. An early approach [45], in which the cerebellar and basal ganglia circuitry was modeled by means of simple feedforward neural networks and combined with the DIRECT-model for motor reaching [46], aimed at explaining the behavioral difference between Parkinsonian patients and controls in a motor adaptation task. According to this model, when learning in the basal ganglia is deactivated to mimic the neurodegeneration of dopaminergic nigrostriatal neurons, continuous erratic movements occur. This compares well to data from patients who show only a crude adaptation. Recently, Caligiore et al. [36] designed a basal ganglia-cerebellar-thalamo-cortical system to explain the development of tics in Tourette. Although the model can recreate changes in the firing rates of cells in animal models of the disease, it does not implement a motor task. Capirchio et al., [47] used a system-level model to simulate a reaching task, which requires to reach three targets from a home position. In this model, the basal ganglia are represented by an actor-critic reinforcement learning account and the cerebellum as a feed-forward perceptron. Lesions to the cerebellum part showed effects observed in patients with cerebellar ataxia. Another recent model by Todorov et al. [48] focused on the role of the cerebellum and basal ganglia in motor adaptation. The basal ganglia implement action selection of a cortical motor program representing a movement trajectory. It is trained by the difference of successive reward prediction errors to support learning when performance improved and suppress the recent action when performance decreases. The cerebellum computes a small correction to the cortical motor program by means of a neural network trained with error backpropagation. In their model, any cerebellum-induced change in performance activates learning in the basal ganglia creating a credit assignment problem about the source of a gain or decline in performance. They therefore propose the existence of a critic somewhere in the brain that determines when each component participates in learning.

Another part of the brain heavily involved in motor execution are the central pattern generators (CPGs) in the brainstem and spinal cord [49–52], that are not only involved in locomotion but also reaching [53–55]. In mice, stimulation of brainstem neurons in the lateral rostral medulla leads to complex forelimb reaching and grasping behavior, where different populations of neurons trigger different patterns of behavior [56]. The large diversity of specialized motor-related neurons in the brainstem integrates information from the cortex, thalamus, cerebellum, and basal ganglia [57]. CPGs became very popular in the research field of neurorobotics leading to sophisticated demonstrations of complex motor actions [58–61]. However, CPGs need some form of more high-level control when recruited for goal-directed behavior.

We introduce here a systems-level computational model that includes the basal ganglia, motor cortex, cerebellum, and brainstem. The focus of our study is the potential division of labor and learning in motor coordination, particularly in reaching and motor adaptation tasks. However, we do not aim to develop a rigorous implementation of neuro-biological details for each subsystem, given the still relatively poor understanding of the neural circuits in these brain parts.

## Results

### Model design

**Fig 1:**
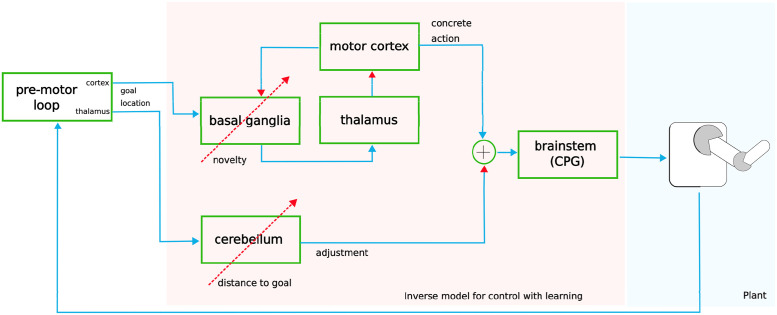
Design of the model. A goal position, that may be determined by the pre-motor cortex-basal ganglia loop, has to be reached. This goal informs both, a motor cortex-basal ganglia loop and the cerebellum. The motor cortex-basal ganglia loop selects a concrete action, which determines the parameters of the CPG in the brainstem. Learning occurs when an achieved hand position is novel through dopamine-modulated Hebbian plasticity that reinforces the association between the executed action and the reached hand position. The cerebellum produces small adjustments to the CPG parameters that reduce the distance between the goal and the achieved position in the current task. Learning occurs through perturbation-based learning using the distance between the goal and the reached position as an error signal.

The model was designed in an open-loop control framework (Fig 1) in order to study its potential and limitations. In an open-loop control framework, the CPGs already provide movement dynamics but need to be under top-down or feedback control [58]. Our plant is a robotic arm with four degrees of freedom. In the reaching task, the shoulder’s yaw, pitch and roll, and the elbow were each controlled by an independent CPG network following the implementation of Nassour et al. [58, 62]. Each CPG network is formed by three layers: a rhythm generation layer that can generate multiple activity patterns, a pattern formation layer that shapes the generated pattern, and a motor neuron layer that drives the joint. While we do not neglect the existence of feedback pathways and closed-loop control, we start here with a model that does not include feedback except for learning. Thus, further upstream motor centers have to provide parameters that manipulate the movement dynamics of the CPG. Our model determines those parameters from two components. The motor cortex-basal ganglia interactions select concrete actions while the cerebellum fine-tunes those actions. The existent network in the brain is of course more complicated. For example, output neurons of the basal ganglia that project to the thalamus have collaterals that target different regions of the brainstem [56]. The term concrete action refers to the observation that movements can be decomposed into a finite set of elementary movements [63] and that activation of the motor cortex produces a limited set of muscle activations [64]. Action selection (BG) and action refinement (cerebellum) are learned through different biologically plausible mechanisms.

A recent hypothesis about the functional structure of the cerebellum is that the recurrent connectivity in the cerebellar cortex implements a reservoir of dynamic activities [65–67] instead of the classically hypothesized feedforward structure. Inputs from the cerebral cortex enter via the mossy fibers a strongly connected recurrent network formed by granule and Golgi cells in the cerebellar cortex, allowing complex patterns to evolve over time even after the inputs have stopped [68]. These spatio-temporal patterns in the reservoir can then be detected by the Purkinje cells to produce appropriate responses [69]. In order to benefit from this dynamical function of the cerebellum, we use the reward-modulated reservoir framework proposed by Miconi [70] as a model of the cerebellum. While the model of [70] is agnostic with respect to localizing the reservoir in any particular area of the brain, it has been used to control a musculoskeletal model of the human arm with four degrees of freedom and 16 muscles in a reaching task with two fixed targets. The reservoir learns by means of a perturbation learning rule, where random perturbations are individually applied to the neurons of the reservoir with varying amplitude and fixed frequency during a trial. At the end of a trial, the reached location is compared to the intended location to compute an aiming error signal. Depending on whether this error decreased or increased compared to the last similar trial, the weights inside the recurrent network are adapted depending on the occurrence of a perturbation (which is maintained by an eligibility trace) and the improvement or worsening of the aiming error. Perturbation learning is an alternative to error backpropagation and is considered more biologically plausible as all computations are local to the neurons.

Although the reservoir network of [70] is not related to the particular structure of the cerebellum, its neurons can be divided into two groups, depending on whether they are output cells or not. Following the interpretation of the cerebellum as a reservoir computing machine [66, 67], output neurons would correspond to the Purkinje cells and non-output neurons to the granular and Golgi cells. Cerebellar parallel fibers implement therefore the readout connections, and recurrent connections between granule and Golgi cells provide the necessary dynamic behavior. However, there is no explicit distinction between excitatory granule cells and inhibitory Golgi cells in the version of the model that we use.

The cortex-basal ganglia component is inspired by recent ideas regarding a hierarchical organization of the basal ganglia and cortex [42, 43]. Specifically, we proposed that the brain achieves goal-directed behavior through a cascade of decisions made by the multiple cortico-basal ganglia loops, each creating an intermediate objective at a different abstraction level [44]. Planning starts in the ventral or limbic loop with the desire for a particular internal or external reward known to be achievable given the current state. The dorsomedial or associative domain then determines the state needed to be reached in order to obtain the reward. The desired state is transformed into a motor goal by a further loop, e.g., by moving the hand to a particular location to satisfy the objective of reaching the object. Finally, the motor goal is transformed into a concrete action plan that may be executed by an open loop model, e.g. central pattern generators (CPGs). Let’s summarize the above concept with an example from everyday life: Our limbic system signals the need for water and we decide to reach for a glass of water, which in turn determines the motor goal in form of the spatial coordinates x,y,z, or the corresponding joint angles. The motor cortex-basal ganglia loop will then select a concrete action that moves the arm to the motor goal. The advantage of our hierarchical approach is that the motor goal is task-independent. After a decision about the target object is determined by the premotor loop, the reaching action does not need further information about those decisions made by the earlier loops. As we have already shown how such a set of decisions could be learned by dopamine-modulated plasticity [44, 71], we focus here on the motor loop only and how a motor goal is transformed into a concrete action and its final execution.

We have also recently demonstrated that learning in multiple cortex-basal ganglia loops cannot rely on a single prediction error signal being identical for all loops [44]. While a reward prediction error is well suited for the limbic loop, the motor loops should be trained by different signals to make them specific to the motor content, independent of the planning and motivational aspects of the task. We use here a dopamine response that indicates the novelty of the achieved movement [72].

A further implication of our framework is that the goal location coming from the pre-motor cortex has initially no meaning. The meaning of such internal signals must be first discovered by active exploration via the environmental act-and-sense loop. Learning occurs after the motor action by sensing its outcome—the reached location—in the premotor cortex. Thus, the outcome is linked to the action that leads to the outcome, providing meaning to the goal signals from the premotor cortex. In our motor loop, actions are initially randomly activated and a phasic increase of dopamine indicates the novelty of the achieved movement, modulating plasticity in the motor striatum to connect outcomes to concrete actions. Supported by the ideomotor theory [73–75], we assume that this active exploration via the environmental act-and-sense loop is a necessary step that takes place prior to goal-directed behavior—but may continue during the lifetime—as the brain has initially no representation of the body kinematics (and dynamics).

### Reaching with the cerebellum alone

As a reference, we initially test the reservoir model from Miconi [70] to mimic cerebellar learning. Following the procedure introduced by Miconi, the activity of all cells in the reservoir is randomly initialized to a small value at the beginning of each trial, the corresponding input is set, and the network is simulated for 200 milliseconds. The input is then deactivated and the network relaxes its activity for 200 additional milliseconds. The mean activity in the last 200 milliseconds of the reservoir’s output cells is then transformed linearly into the six parameter values of each CPG layer (4 joints, therefore 24 output values). Thus, the reservoir encodes the values for the full arm movement, i.e. all joints. The network has to learn reaching movements towards 8 different arbitrary targets within the arm’s workspace.

**Fig 2:**
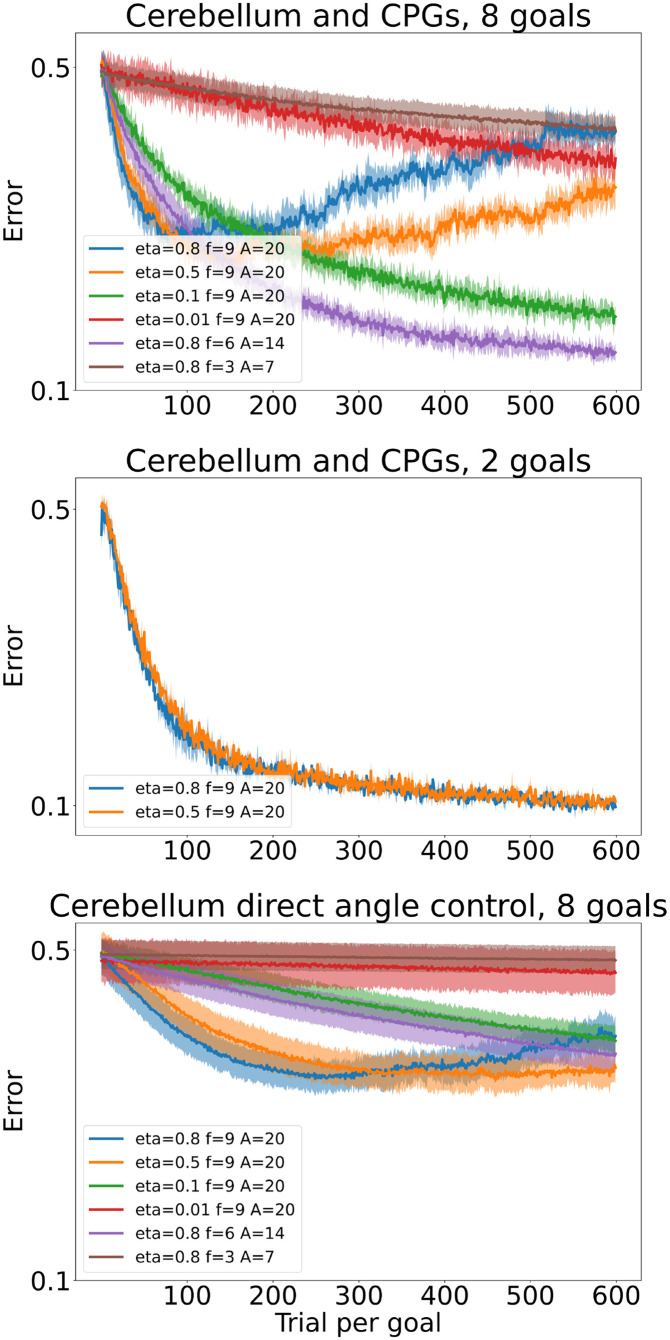
Reservoir’s performance. Performance of the reservoir with different parameter configurations: eta is the learning rate, f is the frequency of the perturbation and A is the amplitude of the perturbation. For each configuration, 50 different simulations are run, each with a different random seed producing different initial conditions, goals, and noise values. On all plots, the Euclidean distance between the goal and reached location over all simulations and including different goals is shown. A: The reservoir sets the parameters of a CPG network controlling each joint. The system is expected to learn 8 goals. Slow-learning networks hardly reduce the error. Fast-learning networks are unstable: They initially appear to learn the task, but then networks tend to forget previous knowledge. B: The same network is used but asked to only learn 2 goals. Configurations that were unstable with 8 goals are stable in this simpler version of the task. C: The output of the reservoir is transformed directly into joint angles (no CPGs are used). The performance of this network is worse than when including the CPGs. Shaded area next to each curve show the standard deviation of the mean.

The perturbation learning rule used in the reservoir depends strongly on three parameters: the learning rate (*η*) or step size, the perturbation frequency (*f*) which determines how often the activity of the cells is perturbed, and the perturbation amplitude (*A*) which determines the size of the perturbation. Therefore, *f* and *A* control the level of noise in the network. Models with a small learning rate or low noise parameters decrease the error only by a small amount (see Fig 2A). Models with intermediate levels of noise or learning rate are able to solve the task but converge to different error levels. Models with faster learning become unstable: the distance to the goal initially decreases, reaches an asymptotic value, and then increases again. The same network configuration does not become unstable in a simpler version of the task in which only 2 goals are required to be learned (see Fig 2B). Results of an exhaustive parameter variation are given in S1 Fig.

On a further control configuration, CPGs are removed and the activity of the reservoir’s output cells is directly linked to the change in the 4 joint angles. Those angles are transformed into a resulting hand position using a kinematic model. Networks with less noise are weaker than those including the CPGs (see Fig 2C). Fast networks become unstable, similarly to the model that includes the CPG. Thus, the CPG component is rather beneficial and does not account for the observed limitation of the reservoir when asking it to learn movements to a larger set of goal locations.

In summary, motor learning by the reservoir alone is sensitive to learning parameters, particularly when multiple target movements are required.

### Reaching with the cerebellum and basal ganglia

**Fig 3:**
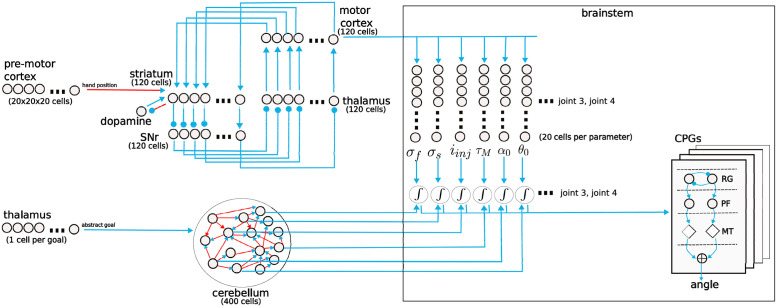
Detailed view on the computational model. Arrows indicate excitatory synaptic connections between neurons. Red arrows indicate plastic connections. Lines ending with a circle indicate inhibitory connections. The closed motor cortex-basal ganglia loop has as many stripes as concrete actions. The direct pathway within the basal ganglia selects one of 120 possible concrete actions. This large number of actions ensures sufficient movement diversity within the reaching space of the arm. Each action is represented in a discrete channel connecting the corresponding cortical, striatal, substantia nigra pars reticulata (SNr), and thalamic cells. Each discrete action activates multiple sets of neurons representing possible CPG parameter values. Each CPG is formed by three layers: RG is the rhythm-generator layer, PF is the pattern formation layer and MT are the motor neurons. The 6 parameters per CPG being adapted are: the time constant *τ*_*m*_, a shape parameter for the current–voltage curve of the fast current *σ*_*f*_, the potassium conductance normalized to the leak conductance *σ*_*s*_ and the injected current *i*_*inj*_ of the rhythm generator neurons of the CPGs. Further, *α*_0_ and *θ*_0_ which are the slope of the sigmoid and the center of the curve of the pattern formation layer of the CPGs. The final parameter value associated with each action is computed by integrating the activity of parameter cells weighted by their preferred parameter value. The cerebellum receives as input an abstract representation of the current goal (no position), one cell per possible goal. In the brain, that position may be encoded within the thalamus of the premotor loop. 24 of the 400 cells in the reservoir project outside (6 parameter values x 4 CPGs) and their activity contributes to the final CPG parameters. Only a single set of neurons for just one CPG is shown in the figure.

In order to test if the division of labor between the basal ganglia and the cerebellum can avoid instabilities, we tested our full neuro-computational model (see Fig 3 for a more detailed view of the model), involving both components, on the same reaching task as before.

The possible concrete actions are encoded by a neural population called the motor cortex, which is part of a motor cortex-basal ganglia loop. Each cortical cell projects to a set of neurons that use a population code to represent the CPG parameter values (see Fig 3). Each cell in these parameter populations is assigned a preferred parameter value. The final parameter value is decoded by computing a sum over the preferred parameter values, weighted by the activity of the corresponding cell. The weights of the connections from the action encoding population to the parameter encoding populations are fixed and random.

The basal ganglia network is a simplified version of our previous model [44, 76, 77], including only a direct pathway (striatum → substantia nigra pars reticulata → thalamus → cortex). Selection occurs when the constant inhibition exerted by the substantia nigra on the thalamus is removed by a corresponding activation in the striatum, allowing a specific cell in the thalamus to get activated and increase the firing rate of the corresponding concrete action. Despite some agreement on the functional role of different basal ganglia pathways there is nevertheless some variability particularly with respect to the indirect and hyperdirect pathway [4]. For the purpose of our study, we only need an intact function of the direct pathway and thus keep the model simple to save computation time. However, more complex motor tasks may benefit from considering additional basal ganglia pathways.

Dopamine-modulated Hebbian learning in the striatum links the input from the goal-encoding cells to the motor program. Novelty-based learning in the basal ganglia works as follows: After every movement, the input activity of the dopamine cell is increased from its baseline to 1, triggering plasticity in striatal neurons. The activity reached by the dopamine cells is however limited by a prediction obtained from the inhibition produced from the striatum, which is also subject to plasticity. The dopamine level reaches its maximum value only when an action is executed for the first time as the striatal inhibition increases after each movement. The same dopamine signal reaches all cells.

Unlike previous action-selection models of the BG, we only implement plasticity between the premotor cortex and the basal ganglia. It is common in computational models to assume that the BG implement a winner-take all mechanism between input action channels [6]. In classical action-selection models, the main inputs to the BG loop are the available actions and the BG must select one of them, usually the most salient one. In those models, the BG does not implement any transformation of the input information, it only removes the less salient action channels. Plasticity is then implemented in the connections within the loop (motor cortex) to assure a proper action selection. Based on our previous models [9, 44, 77, 78], we instead assume that each BG loop learns a goal-response map, which links objectives to appropriate actions. The input to the loop is different than action-selection models as it results from the information processing in previous loops. For selecting concrete actions, plasticity is then required at the projections from the premotor cortex, not necessarily at the projections from the motor cortex.

The cerebellum is modeled as a pool of 400 randomly connected cells. The projections within the pool are plastic and follow a perturbation-based learning rule [70]. 24 of those 400 project outside (6 parameters per joint). The activity of these output cells is added to the parameter value encoded in the parameter cells before they are set in the CPGs.

**Fig 4:**
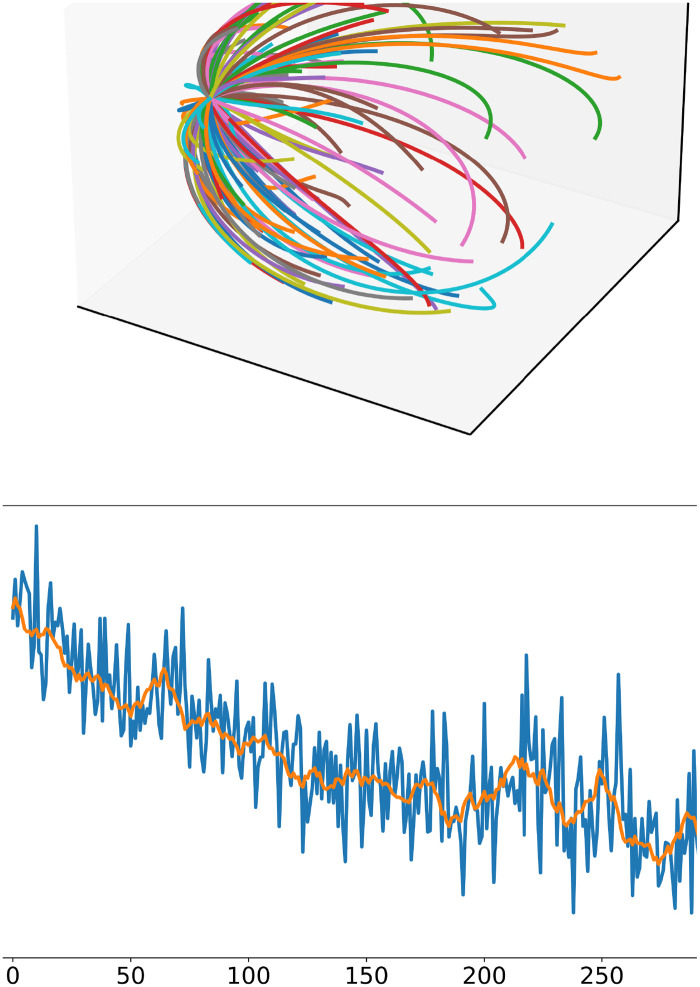
Basal ganglia training. The initial training of the basal ganglia is performed by randomly activating desired outcomes. A: Learned trajectories of 120 concrete actions of an example simulation. Each of the 120 lines in the plot represents the trajectory of the hand after selecting one action starting from the same position in one simulation. The basal ganglia can therefore select one among 120 trajectories. B: Result of learning in the basal ganglia by exploration via the environmental act-and-sense loop. At the beginning of every training trial, a random goal (desired hand position) is activated. Then, if no action cell had a strong enough firing rate, a random action is activated by setting its activity to 1. The basal ganglia learn to map the reached position with the activated action. Thus, learning associates the outcome with the action that leads to the outcome (act-and-sense). The plot shows that, over time, intended outcomes become associated with an action that closely reaches it. The blue line represents the mean distance over 50 simulations and the orange line is the average of the mean distance with a time window of 10 trials.

The basal ganglia are trained prior to the task simulation until the model replicates a randomly selected outcome for three times in a row. The main goal of this process is for the basal ganglia to create a map between outcomes (final hand positions) and concrete actions. During training, 120 actions are activated randomly, the outcome is observed and finally the association strength between the outcome and the action is increased. This creates a meaning for the pre-motor cortex neurons, which do not have one until activated by an observation. On each simulation a different set of 120 actions are defined, each associated with a random set of CPG parameters. Later, the outcome-action map is be used to select an action based on a desired outcome (Fig 4). The BG therefore are not trained on the goals of the task, but develop knowledge about the possible actions to choose from. Activity of the BG during an example trial is shown in S2 Fig.

**Fig 5:**
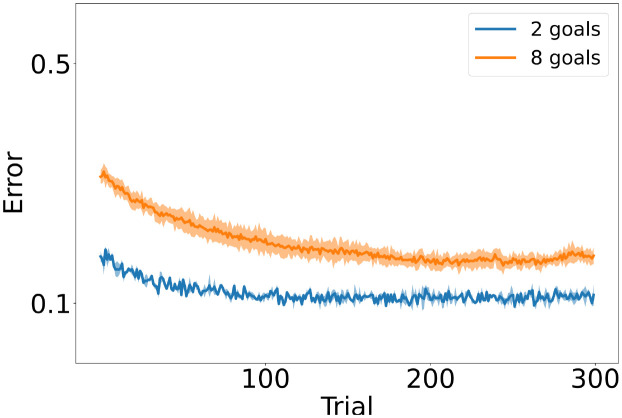
Training the full model in a reaching task. The full model includes a cortex-basal ganglia component that has been pretrained to allow the selection of a concrete action to a given arbitrary goal. The full model is then tested with either 2 or 8 random goal positions and required to learn to execute a movement to the given goal. The full model is not unstable when the number of goals is increased from 2 to 8. The shaded area next to each curve shows the standard deviation.

We simulated the same reaching task with 2 and 8 goals. We used in the cerebellum a learning rate *η* = 0.8 and noise parameters *f* = 9 and *A* = 20. These parameters correspond to a fast network, which produced an unstable behavior when learning the task directly. Our simulations show that, with the full model, both tasks can be learned without any problem of stability. The reason is that learning is simpler as the BG introduce an initial solution through a concrete action and only small adjustments are produced by the cerebellum (see Fig 5). Not surprisingly, learning is also much faster than with the cerebellum alone.

### Visuomotor adaptation task

After demonstrating the model’s basic functionality, we now investigate its ability to explain observations in motor adaptation. Motor adaptation refers to a particular type of motor learning in which a well-known action is modified to maintain performance after a change in the environment or the body [79]. One common way to study adaptation in an experimental setting is to impose a visuomotor rotation [80]. In such experiments, participants are seated in front of a screen and are required to move a cursor toward a target location with a straight inward-outward movement [81]. The cursor is not visible throughout the whole trajectory. During the movement, the cursor remains initially at its starting position and then indicates the movement reversal point. Thus, subjects only obtain visual feedback about their movement outcome with respect to its endpoint. After several baseline trials, the cursor’s coordinate system is rotated with respect to the coordinate system of the hand movement space. As participants are not informed about the manipulation and only observe the outcome, they slowly alter their behavior to cope with this perturbation. Errors are reduced trial by trial suggesting that it is controlled by an implicit learning process. Once the perturbation is removed, an aftereffect is observed: The participants initially overcompensate and then slowly, trial by trial, return to normal movements [80]. However, when participants were instructed about the nature of the perturbation and an instruction to compensate for it, they immediately applied it and had almost no error in the trial after the information has been given [80].

We confront our model with the visuomotor adaptation task used by Mazzoni and Krakauer [80]. After initial training on the baseline trials on two random goals, the coordinate system of the cursor is rotated by 45 degrees. As with the participants, we have three types of model simulations: in a first simulation, the model receives no information about the perturbation (rotation group); in a second simulation, the model is forced to adopt an explicit cognitive strategy by instructing it to direct the movement 45 degrees counterclockwise (rotation + strategy group); and in a third simulation the model is also instructed to direct the movement 45 degrees counterclockwise but the cursor is not perturbed (strategy group).

**Fig 6:**
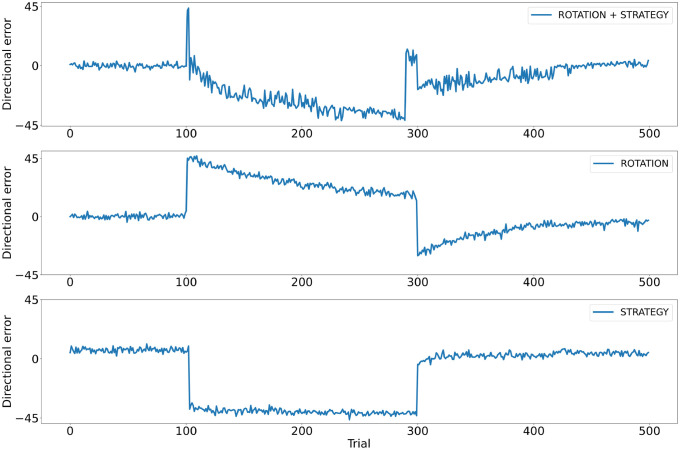
Visuomotor adaptation. Test of the model with a visuomotor rotation task [80]. After initial training on baseline trials, the coordinate system of the cursor is rotated by 45 degrees. Then, after 200 trials interacting in the perturbed environment, the conditions return to the baseline. The first row shows the performance of models that, after 2 trials in the perturbed experiments, are informed about the perturbation by changing the goal location and re-setting the goal location later on (ROTATION + STRATEGY). The second row shows models that are not informed about the perturbation (ROTATION). The third row shows models that are provided with the new goal, but the environment was not perturbed (STRATEGY).

The perturbation is simulated in our model by rotating the final outcome of the hand movement by 45 degrees, as also human subjects have no visual feedback of their arm trajectory. Thus, after the rotation is introduced, the models make a 45 degree error (in Fig 6 at trial 100). The manipulation leads to an error signal in the cerebellum, which shows a strong increase once the rotation is introduced, but it does not induce novelty-based learning in the BG. In the strategy condition, the model is instructed to counter the perturbation, as with human subjects in the original experiment of Mazzoni and Krakauer. The instruction to counter the perturbation is given to our model as a change in the goal represented in the premotor cortex. The new goal corresponds to a position rotated from 45 degrees with respect to the initial one. The new input triggers the BG to select a different concrete action, one that moves the arm closer to the new goal direction. As with the participants, the instructed model immediately reduces the error close to zero (trial 103 in the Fig 6). This rapid change in movement direction, similarly to what was observed in humans, is in our model proposed by action selection at the BG level, as the cerebellum outputs only gradual corrections and requires multiple repetitions to adapt. In the following trials, the new motor goal is maintained and therefore the basal ganglia continues selecting the same concrete action. The change in the motor goal due to the instruction also affects the error computed at the level of the cerebellum, as the observed position of the pointer is compared to the intended motor outcome (aiming error, not task error). Importantly, as observed in human subjects, this explains why the model shows increasingly large directional errors over the following trials, over-adapting to the perturbation.

In the original experiment of [80], after over-adapting to the perturbation, participants were instructed to stop using the explicit strategy. We give our model this information by a change in the goal, setting it back to the initial position, changing therefore again the concrete action, and as a consequence the error at the cerebellum. The new concrete action produces an immediate change in the direction, as observed in humans (see the increase in the error in Fig 6 rotation+strategy group around trial 300). When the perturbation is finally removed (10 trials after the last instruction), models and subjects show an after-effect and the error slowly declines. During this last period there is no further change in the motor goal and the corrections are therefore only produced by the cerebellum.

Our simulations of the rotation group (no instruction) show no immediate direction change. Like the human subjects, the model slowly adapts to the perturbation reducing the error trial by trial. Once the perturbation is removed, an aftereffect is again observed: A change in the direction of the error and a slow return to zero.

The simulations of the group that was instructed, but not perturbed, show no slow change in the error and no aftereffect. The change in the concrete action moves the arm toward the new desired direction and only very small changes are introduced by the cerebellum, as errors are computed according to the new instructed motor goal (aiming error). Thus, no after-effect occurs, similar to the data from human subjects. A comparison of the error signal in the Cerebellum under the three conditions can be observed in S3 Fig.

When we remove the cerebellum such that it provides no contribution to the CPG, in the rotation+strategy condition the BG compensates for the perturbation and the over-adaptation observed in the full model does not occur (S4 Fig).

Concluding, our model can replicate the main properties of the data of [80]. However, we spotted also small differences such that the model’s implicit learning process is slower than those of the participants. This could be because in the experiment of Mazzoni and Krakauer, the subjects were expected to make wrist movements of only 2.2cm, much shorter than in our setup.

### Motor variability

Although motor variability has been often considered an undesired characteristic that should be avoided, it has been shown that task variability is a good predictor of individual learning ability [82–84]. Greater task-relevant variability predicts faster learning.

In our model, learning in the cerebellum depends on perturbations to the activity of the cells and requires appropriate noise levels. In the reservoir, noise is defined by two parameters: the frequency by which a perturbation is introduced into the activity of the cells and the amplitude of this perturbation.

**Fig 7:**
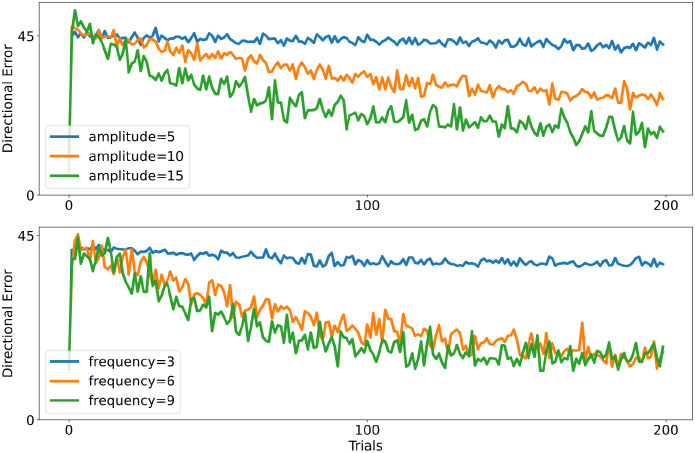
Variability in the visuomotor adaptation task. Higher levels of noise produce faster adaptation until a particular noise level is reached. The plot on the top shows the performance of models with different perturbation amplitudes. The plot below shows the performance of models with different perturbation frequencies.

We compare models with different frequencies and amplitudes in the same perturbation task used in the previous section. Models with higher noise amplitude adapt faster to the rotated environment (see Fig 7 top). Increasing the noise frequency also allows a faster adaptation (see Fig 7 below). However, changes in the learning speed saturate at sufficiently large values: the learning speed is not further improving when the frequency level is increased. This compares well with the observations of van der Vliet et al. [82]

## Discussion

Our computational model is meant to advance the ongoing discussion on the contribution of the basal ganglia and cerebellum to motor learning. In the 3D-reaching task, we demonstrate the benefit of the concrete action selection by the basal ganglia, compared to a cerebellum-only model. Combined with the basal ganglia, the cerebellum is now only required to fine-tune the motor parameters, but not to learn and store all parameters of the arm movement. This further agrees with the super-learning hypothesis [33], as both learning systems interact in a pipeline organization: with the cerebellum using the results of the BG. Simulations with the full network are able to reach a good performance with parameter values that produced unstable behavior in an isolated Cerebellum model.

Of course, this advantage depends a lot on the assumed complexity of computation localized in the cerebellum and on the complexity of the control architecture. While we have used an open loop control and a target endpoint, models from the neuro-robotics community (e.g. [26, 85, 86]) typically use feedback control, which ensures that the desired endpoint will be reached, while a trajectory planner sets up the desired joint angles and the according velocities. In those approaches, models representing the cerebellum are embedded in the circuitry as forward and inverse models, and help to bring the actual trajectory closer to the desired trajectory. However, references to the basal ganglia in those studies are rather abstract and no explicit models of the basal ganglia have been used to solve robotic motor-control tasks. Demonstrating our model in the motor reaching task is meant as a proof of concept, but not to compete with state-of-the-art robotic solutions.

Adaptation tasks that include an additional cognitive strategy to counter the error [80] provide an interesting test scenario for our model. When human subjects are informed to use a strategy to overcome an error due to a rotational bias, they nevertheless continue adapting, leading to increased errors, although the strategy was effective and the task could have been done without error. In our model, the cognitive strategy affects the motor goal encoded in the premotor cortex and as a result, a different concrete action in the basal ganglia is selected to compensate for the rotational bias. However, although the cognitive strategy works fine for the task, the cursor endpoint is not consistent with the motor goal, which leads to continuous adaptation and to an increasingly bad performance on the task. This clearly shows that motor adaptation depends on an error signal that uses a motor goal (presumably defined in sensory space) but not a task goal. However, recent studies showed that under conditions where the sensory prediction error is non-zero, the task error can also have an influence [87] and both errors may interact with each other, presumably within the cerebellum [88].

The error used for cerebellar learning can be computed in different ways. It may be computed by comparing the predicted sensory consequences of the planned motor action with the outcome, i.e. sensory feedback, see also [20]. Alternatively, the motor goal [89] may already be defined in sensory space (cursor at an intended location) and the executed action is selected to reach this goal. Our approach follows this direct updating account without the need to use a forward model for computing a sensory prediction error. Recently, similar ideas have been put forward and the latter approach has been formulated as direct policy updating [90] and compared to the traditional framework according to which a forward model is updated and inverted for motor control. There is an ongoing debate about the need for motor-based forward models beyond the own body if error signals can be obtained by alternative action-outcome frameworks [91].

A critical assumption of our model is novelty-based learning in the BG. Traditionally, BG models use reward prediction errors as a model of dopaminergic signalling, where reward is linked to the task performance. However, there is evidence that dopamine neurons encode multiple signals and that different types of dopaminergic cells are connected with distinct brain networks [92]. Many cells fire to non-rewarding events [72]. Thus, motor learning may not be directly driven by a signal following task performance. Novelty signals allow the basal ganglia to acquire knowledge that is task-independent, reducing catastrophic forgetting. In our model, synaptic plasticity follows a 3-factor learning rule, with dopamine as the third factor. The size of a phasic increase in the dopamine signal depends on the prediction computed on basis of the activation of striatal neurons. With repetitions of the same action, the prediction increases and thus the dopamine signal decreases. As the dopamine signal depends on an internal context, here the activation of striatal neurons, it allows, in principle, learning of different tasks independent of childhood experience. However, we consider our novelty-based learning being a comparably simple implementation of this interesting field of research.

Taylor and Ivry [93] designed a mathematical setpoint state space model to replicate the data of [80]. The model includes a learning equation to calculate the current internal estimate of the rotation. Different from previous approaches using similar techniques to model other adaptation protocols, their equations include a representation of an explicit strategy. Its biological implementation, however, is unclear and no reference to action selection or the basal ganglia has been made.

Motor adaptation, but not particularly the role of cognitive strategies, has also been modeled by Todorov and colleagues [48] using a model of the basal ganglia and cerebellum. In addition to several differences at the implementation level, there are noticeable differences at the conceptual level of the model design that shall be discussed. According to their model, both the cerebellum and basal ganglia aim to counteract the perturbation. The cerebellum uses the error between the movement endpoint and the target to compute a correction of the motor program. Different from our approach where the basal ganglia are trained by a novelty learning rule, their basal ganglia model is trained by a temporal difference of the movement error, indicating an increased or decreased success on the task. Due to conflicts in the adaptation process, they created a critique that implements an arbitrator which controls when adaptation should be led by the basal ganglia and when by the cerebellum.

In our model, the basal ganglia select a motor action that is under strategic control. For example, to move a cursor upwards, it can choose to move the hand in a different direction. We have proposed a cognitive-to-motor hierarchy that can convert a task goal into a motor goal and the choice of the particular action [44], while we here only modeled the motor selection part. At the motor level, learning in the basal ganglia should not follow a task-performance reinforcement signal, but rather a motor-performance signal. In the present study, based on the heterogeneity of the dopamine system [94], we decided to learn on basis of a novelty learning rule in the basal ganglia. If the achieved position after a cerebellar correction is similar to positions observed during the initial training, then no learning will occur in the basal ganglia and therefore no conflict between the basal ganglia and the cerebellum occurs. Further, even if the position is new, learning will occur according to the achieved position and not the current motor goal, producing no conflict in the following trials.

The adaptation experiment we simulated includes an explicit instruction which produces an immediate reduction in the error. We represented this as a change in the motor goal which allows the BG to select a new concrete action, changing instantaneously the simulated movement direction. In comparison, the BG in the motor adaptation model of Todorov and colleagues [48] learns by means of a temporal difference of the task-performance between the current and previous trials and thus, adapts slowly and requires an exploration period after the perturbation is introduced to find the appropriate correction. In order to simulate an explicit strategy, the model of Todorov et al. would need to include an additional mechanism. Further, forcing the BG to learn on task-performance will counteract the learning in the cerebellum, which rather predicts against an ongoing adaptation towards larger task errors as observed in human subjects in the strategy condition.

### Limitations

Models for understanding motor behavior and motor learning can cover many different disciplines. They may include aspects of computational neuroscience, neurorobotics, artificial neural networks, learning rules, and control theory. From each particular viewpoint, present models have limitations, due to the complex nature of the research topic. We aimed for a systems-level design to study the share of labor of different parts performing a simple robotic task and an experimental task in motor adaptation. Of course, each of our model components abstracts a lot from the brain area it shall represent. Our model of the basal ganglia covers some aspects of computational neuroscience and has been previously studied a lot and compared to experimental data [9, 44, 71, 76, 77], although here we only considered the direct pathway of the basal ganglia. The model of the CPG is biologically well-motivated, but more directed at a functional level for neurorobotics [58, 62]. The model of the cerebellum is quite abstract from its biological counterpart and is modeled as a reservoir with perturbation learning, thus avoiding the backpropagation learning rule. It is now also known that basal ganglia and cerebellum are not largely independent of each other but interconnected [95]. Through such direct projections, adaptations learned by the cerebellum could be transmitted to the basal ganglia which could then guide a learning process that incorporates them into the concrete action. Here, we do not consider any direct connection between those structures but simply add their output before setting the parameters of the joints.

The model’s motor cortex is not well motivated on the basis of physiological data but is limited to the idea of representing compact actions. Further, our motor cortex only includes fixed connections. Plasticity is known to occur in the motor cortex and is critical for the development of complex behaviors [96, 97]. In our model, plasticity in the motor cortex could help to optimize the set of actions available to the basal ganglia. For example, parameter refinements learned by the cerebellum could be then incorporated into the cortical representations of the corresponding concrete action. It has been already suggested that sensorimotor knowledge could be exported from the cerebellum to the cortex [98, 99].

Our model does not add much to the field of control theory and to its already sophisticated models of closed-loop control, as we have taken an open-loop approach. However, our approach may be extended to test theories of intermittent control which aim to describe control tasks by serial ballistic movements [100, 101]. The motor tasks we modeled do not pose a challenge to the neurorobotics community. However, a better understanding of the potential contribution of different brain parts can be helpful for designing more sophisticated robots, particularly with respect to the division of labor between cortical areas, basal ganglia, and the cerebellum.

We have also related our model with data suggesting that noise is beneficial for learning [83]. As observed in behavioral experiments, higher variability leads to faster learning. We need however to be careful with these observations, as planning noise needs to be differentiated from execution noise [82]. Our model only includes planning noise, which is represented by small perturbations of the activity of cerebellar cells, but does not include execution noise which could be produced at the level of the muscles and independent of the high-level signal reaching the joints. In our simplified implementation, the same high-level signal will produce always the same movement, something that may not happen in a more realistic environment. The relation between planning and execution noise, and the linked credit assignment problem, are topics for future studies. Further, there is evidence that the nervous system can regulate variability according to the context [102]. Increasing reward probabilities can reduce movement variability while decreasing reward probabilities produce the contrary effect [103].

Our model has not been compared to human kinematic data as other previous approaches based on reinforcement learning [104]. All simulations shown here use random actions to highlight that the model can learn to use any type of movements.

We should emphasize here that at the present stage our results are limited to a proof of concept. In order to accept the hypothesis presented here, more experiments are required and a proper comparison to other models of the basal ganglia—cerebellum network are necessary. Further, for now only a qualitative comparison with experimental data is presented.

### Conclusion

Brainstem circuits are highly specialized centers for motor control which are informed by more upstream centers such as the motor cortex, thalamus, basal ganglia, and cerebellum [57]. How central pattern generators (CPGs) are influenced by basal ganglia and cerebellar sub-systems has been the central aim of our model design. We propose that cortex-basal ganglia loops select concrete actions that can be fine-tuned by the cerebellum. While the traditional view links learning in the basal ganglia to reward-based learning, and in the cerebellum to supervised learning, our approach suggests that learning in the basal ganglia is not uniform, but rather depends on the origin of the cortex—basal ganglia loop [44]. While the limbic basal ganglia are well suited for learning about the success of the task, the motor basal ganglia shall rather consider aspects of motor execution, such as a novelty-based dopamine signal. This dissociation of labor allows us to explain the surprising observation that human subjects continue to adapt in motor adaptation tasks, although they perform the task without error. In our model, the basal ganglia can counteract the perturbation in motor adaptation by a cognitive strategy. However, as the cerebellum learns about the difference between the intended position and the final arm position, it further contributes to adaptation.

## Materials and methods

### Central pattern generator

Each CPG network is composed of three layers: rhythm-generation neurons, pattern formation neurons, and motor neurons. More details about its neurophysiological basis can be found in [62].

The rhythm-generator layer is composed of two cells that can generate self-rhythms. The membrane potential (*V*) of these cells is defined by:

$$\begin{array}{c} \begin{array}{cc} \tau_{m}\frac{dV}{dt} & =-(V-A_{f}tanh\left(\left(\sigma_{f}/A_{f}\right)V\right)+q-i_{inj})\\ \tau_{s}\frac{dq}{dt} & =-q+\sigma_{s}\left(V-E_{s}\right) \end{array} \end{array}$$ (1)

where *τ*_*m*_ and *τ*_*s*_ are time constants, *i*_*inj*_ is the injected current, q is the lumped slow current, *σ*_*s*_ is the potassium conductance normalized to the leak conductance, *σ*_*f*_ is a dimensionless shape parameter for the current–voltage curve of the fast current and *A*_*f*_ is the width of the N shape of the fast current.

Pattern formation neurons are modulated by the rhythm-generator neurons and by sensory neurons encoding the current joint angles. The activation function is defined by:

$$\begin{array}{c} \begin{array}{cc} PF & =\frac{1}{1+e^{\alpha_{0}\alpha_{PF}\left(\left(\theta_{0}+\theta_{PF}\right)-I_{PF}\right)}}\\ I_{PF} & =\frac{W_{rg}RG+\sum_{j=1}^{n}W_{j}S_{j}}{n+1} \end{array} \end{array}$$ (2)

where *RG* is the activation of the rhythm generator neurons, *W*_*rg*_ is the weight for the connection from the rhythm generator neuron, *S*_*j*_ is the activity of the sensory neurons and *W*_*j*_ the weight of the connections from the sensory neurons. *α*_*PF*_ is a descending control signal that modulates the activity of pattern formation cells and *θ*_0_ is the center of the sigmoid function that controls the balance between the extensor and the flexor.

Motor neurons are defined by:

$$\begin{array}{c} \begin{array}{cc} MN & =\frac{1}{1+e^{5(0.5-I_{MN})}}\\ I_{MN} & =\frac{W_{pf}PF+\sum_{k=1}^{n}W_{k}S_{k}}{n+1} \end{array} \end{array}$$ (3)

The final joint angle (*U*) is obtained by combining the extensor and flexor motor commands:

$$\begin{array}{c} U=Amp\left(MNE-MNF\right)+U_{ref} \end{array}$$ (4)

where *Amp* is an amplification factor, *MNF* and *MNE* are the flexor and the extensor motor neurons activation. *U*_*ref*_ is the joint reference angle.

The parameters *τ*_*m*_, *σ*_*f*_, *σ*_*s*_, *i*_*inj*_ of the rhythm generator neurons and the parameters *α*_0_,*θ*_0_ the pattern formation neurons of all CPGs are set as a results of the BG and cerebellum interactions. The value for the fixed parameters are shown on Table 1.

**Table 1:** Fixed parameter values for the CPGs.

Parameter	Value
*A*_*f*_	5.0
*τ*_*s*_	20*xτ*_*m*_
*α*_*PF*_	1
*θ*_*PF*_	0
*W*_*rg*_	1
*W*_*pf*_	1
*Amp*	5.0

### Basal ganglia

The firing rate of neurons in the basal ganglia is defined by the following equation:

$$\begin{array}{c} \begin{array}{cc} \tau \frac{dmp_{j}}{dt}+mp_{j} & =\sum_{i\in N_{e}}w_{ij}r_{i}-\sum_{i\in N_{i}}w_{ij}r_{i}+B+\epsilon_{j},\\ r_{j} & ={\left(mp_{j}\right)}^{+}. \end{array} \end{array}$$ (5)

where *mp*_*j*_ is the membrane potential, *r*_*j*_ is the firing rate, *τ* is a time constant, *w*_*ij*_ is the weight between the presynaptic neuron i and postsynaptic neuron j, *N*_*e*_ is the group of cells that have an excitatory projection to neuron j, *N*_*i*_ is the group of cells that have an inhibitory projection to neuron j, B is a baseline value and *ϵ*_*j*_ is a noise term drawn from a uniform distribution. ()^+^ converts negative numbers to 0.

Plasticity in the cortico-striatal projection follows the learning rule:

$$\begin{array}{c} \tau_{w}\frac{dw_{ij}}{dt}=f_{DA}\left(DA\left(t\right)-B_{DA}\right)C_{ij}-\alpha_{j}{\left(r_{j}-{\bar{r}}_{POST}\right)}^{2} \end{array}$$ (6)

where *w*_*ij*_ is the weight between cortical cell i and striatal cell j, *f*_*DA*_(*DA*(*t*) − *B*_*DA*_) is the dopamine modulation which depends on a phasic change between the current dopamine level (*DA*(*t*)) and the baseline dopamine level (*B*_*DA*_), *C*_*ij*_ is the correlation between cortical cell i and striatal cell j and $\alpha_{j}{\left(r_{j}-{\bar{r}}_{POST}\right)}^{2}$ is a normalization term that limits the weight growth.

Based on biological findings [105, 106], a phasic increase in dopamine (*DA*(*t*) > *B*_*DA*_) strengthens the weights between active neurons while a phasic decrease (*DA*(*t*) < *B*_*DA*_) reduce their value. The function *f*_*D*_*A*(*x*) controls the rate of increase and decrease and takes values *Kb* for positive *x* and *Kd* for negative *x*.

The correlation term (*C*_*ij*_) is computed following the equation:

$$\begin{array}{c} C_{ij}=\left(r_{i}-{\bar{r}}_{PRE}-\gamma_{PRE}\right){\left(r_{j}-{\bar{r}}_{POST}-\gamma_{POST}\right)}^{+} \end{array}$$ (7)

where *r*_*i*_ and *r*_*j*_ are the firing rates of cortical cell *i* and *j*, *r*_*PRE*_ is the mean firing rate of the cortical population and *r*_*POST*_ is the mean firing rate of the striatal population, *γ*_*PRE*_ and *γ*_*POST*_ are thresholds.

The dopamine level *DA*(*t*) is computed following the activity of a cell whose activity is governed by:

$$\begin{array}{c} \tau \frac{DA(t)}{dt}+DA\left(t\right)=P\left(t\right)\left(1.0-\sum_{i\in StrD1}w_{ij}^{DA}r_{i}\right)+B_{DA} \end{array}$$ (8)

where *B*_*DA*_ is the baseline dopamine level, *P*(*t*) controls that dopamine changes are produced only after a movement is executed, being 1 after a movement and 0 otherwise. The dopamine level is inhibited through direct striatal connections with weights $w_{ij}^{DA}$.

Projections from the striatum to the dopaminergic cell are plastic and governed by the following rule:

$$\begin{array}{c} \tau_{w}\cdot \frac{dw_{ij}^{DA}\left(t\right)}{dt}=3\left(DA\left(t\right)-B_{DA}\right)\cdot {\left(r_{i}\left(t\right)-{\bar{r}}_{PRE}\right)}^{+} \end{array}$$ (9)

All fixed parameter values are shown in Table 2.

**Table 2:** Values for the fixed parameters of the basal ganglia.

Parameter	Value
*τ*	10
*w* striatum-snr	0.8
*w* snr—thalamus	0.6
*w* thalamus—cortex	1.0
*w* cortex—striatum	0.5
*B* striatum	0
*B* snr	1.1
*B* thalamus	0.9
*B*_*DA*_	0.1
*γ*_*PRE*_	0
*γ*_*POST*_	0.1

### Cerebellum

The cerebellum module follows the reservoir computing framework proposed by [70]. It is composed of 400 neurons with a firing rate *r*_*i*_(*t*) given by:

$$\begin{array}{c} \begin{array}{cc} \tau \frac{dx_{i}}{dt} & =-x_{i}\left(t\right)+\sum J_{ij}r_{j}\left(t\right)+\sum B_{ik}u_{k}\left(t\right)\\ r_{i}\left(t\right) & =tanh(x_{i}\left(t\right)) \end{array} \end{array}$$ (10)

where *J*_*ij*_ are plastic local weights, *u*_*k*_(*t*) is the activity of the goal encoding cells, which is 1 if goal *k* is currently active and 0 otherwise, and *B*_*ik*_ are random weights drawn from a uniform distribution between -0.2 and 0.2.

At every time step the value of *x*_*i*_(*t*) is perturbed with a probability *f*. Perturbations are introduced by adding to *x* a random value drawn from a uniform distribution between −*A* and *A*.

The learning rule depends on an eligibility trace given by:

$$\begin{array}{c} e_{ij}\left(t\right)=e_{ij}\left(t-1\right)+{\left(r_{j}\left(t-1\right)\cdot \left(x_{i}\left(t\right)-\bar{x_{i}}\right)\right)}^{3} \end{array}$$ (11)

The weight change (Δ*J*) is then defined as:

$$\begin{array}{c} \Delta J_{ij}=-\eta e_{ij}\bar{E}\left(E-\bar{E}\right) \end{array}$$ (12)

where E is the error in the current trial and $\bar{E}$ is the mean error. The initial value of the weights *J*_*ij*_ are drawn from a normal distribution with a mean of 0 and a standard deviation of 0.05.

### Kinematic model

The position of the wrist (*x*) given the output joint angles of the CPGs is computed by performing a set of matrix operations following the simple kinematic of the humanoid robot James [107, 108]. This provides us with a fast transformation from angles to hand position.

$$\begin{array}{c} \begin{array}{cc} G_{34} & =[\begin{array}{cccc} cos(\frac{\pi }{2}+elbow) & -sin(\frac{\pi }{2}+elbow) & 0 & 0.16cos(\frac{\pi }{2}+elbow)\\ sin(\frac{\pi }{2}+elbow) & cos(\frac{\pi }{2}+elbow) & 0 & 0.16sin(\frac{\pi }{2}+elbow)\\ 0 & 0 & 1 & 0\\ 0 & 0 & 0 & 1 \end{array}]\\ G_{23} & =[\begin{array}{cccc} cos(\frac{\pi }{2}+roll) & 0 & sin(\frac{\pi }{2}+roll) & 0\\ sin(\frac{\pi }{2}+roll) & 0 & -cos(\frac{\pi }{2}+roll) & 0\\ 0 & 1 & 0 & 0.22\\ 0 & 0 & 0 & 1 \end{array}]\\ G_{12} & =[\begin{array}{cccc} cos(\frac{\pi }{2}+yaw) & 0 & -sin(\frac{\pi }{2}+yaw) & 0.05cos(\frac{\pi }{2}+yaw)\\ sin(\frac{\pi }{2}+yaw) & 0 & cos(\frac{\pi }{2}+yaw) & 0.05sin(\frac{\pi }{2}+yaw)\\ 0 & -1 & 0 & 0\\ 0 & 0 & 0 & 1 \end{array}]\\ G_{01} & =[\begin{array}{cccc} cos(pitch) & 0 & -sin(pitch) & 0\\ sin(pitch) & 0 & cos(pitch) & 0\\ 0 & -1 & 0 & 0\\ 0 & 0 & 0 & 1 \end{array}]\\ G_{02} & =G_{01}\times G_{12}\\ G_{03} & =G_{02}\times G_{23}\\ G_{04} & =G_{03}\times G_{34}\\ x=G_{04}\times [\begin{array}{c} 0\\ 0\\ 0\\ 1 \end{array}] \end{array} \end{array}$$

where x is the position of the wrist, *elbow* is the angle of the elbow joint in radians, *roll* is the angle of the shoulder roll joint in radians, *yaw* is the angle of the shoulder yaw joint in radians, and *pitch* is the angle of the shoulder pitch joint also in radians.

### Training and task simulation details

For the simulations in the reaching task, goals are selected by adding a random number of degrees to the initial arm configuration and then computing the hand position. This ensures that goals are reachable. Only goals that are at a minimum distance of 0.5 from the initial hand position are considered to avoid very short movements.

Every simulation starts with a basal ganglia training block. At the beginning of each trial of this block, the network is simulated with no inputs for enough time to allow it to return to its baseline activity. Then, a random goal is generated and the baseline of the cortical input cells changes according to a Gaussian function with the difference between the cell’s preferred position and the goal. The network is then simulated for 200ms and the activity in the motor cortex is observed. If the maximum activity in the motor cortex is less than 0.05, a random concrete action is selected and the activity of the corresponding action cell is set to 1. If the maximum activity is larger than 0.05, the activity of the most active concrete action is set to 1. Then, an additional 150ms are simulated to allow the parameter encoding cells to reach a stable activity pattern.

Parameter values are then computed by reading the activity of the parameter encoding cells. A sum over the activity of the cells is computed, weighted by the cells’ preferred parameter value. The values for *σ*_*f*_, *σ*_*s*_ are limited between 5 and 10, *i*_*inj*_ is limited between -4 and 4, *τ*_*M*_ is limited between 5 and 15, *α*_0_ and *θ*_0_ are limited between 0.001 and 2.

A movement is executed by solving the CPG equations and transforming the final angles into a hand position using the kinematic model. The baseline of the input cortical cells is then changed according to this new position and the model is further simulated for 100ms. Finally, the baseline of the dopamine cell is increased to 1.0 to allow learning, and a final 100ms is simulated. The activity of the dopamine cells during this period is further restricted through striatal inhibition.

In simulations with 8 goals, the simulation speed is increased by computing the concrete action for each goal in advance. After the initial basal ganglia training, 8 additional trials are simulated, each with one of the goals that will be used later during the task simulation. The concrete action selected and the corresponding parameter values are saved for future use. Then, during the task simulation, the output values of the cerebellum are added to the saved concrete action values.

On every trial during the task simulation, the activity of the cerebellum cells is initially set to a uniform random value between -0.01 and 0.01. Then the corresponding input cell is activated and the network is simulated for 200ms. The input is then turned off and an additional 200ms is simulated. The mean of the activity of the output cell during this final period is considered as the output of the network and added to the parameters obtained through the concrete action. This process was used originally by Miconi [70].

After executing the movement, the Euclidean distance between the goal and the achieved position is computed and used as an error function to train the reservoir. The mean error considered in the learning rule is computed independently for every goal.

In visuomotor rotation paradigms, normally only 2-dimensional movements on a plane are allowed by fixing the arm accordingly. Rotations are introduced according to this two-dimensional plane. As our model normally produces three-dimensional movements we defined the plane according to which the position will be rotated. To solve this problem, we first train the model to reach 2 goals as in the reaching task. Then, during the perturbed period, the final hand position computed with the kinematic model is rotated by a fixed amount of degrees around the axis formed by the vector resulting from the cross-product between the two goals used during training. Angular errors are computed by first projecting the initial and final hand position to the same plane and determining the angle formed by the final position, the initial position, and the goal. Small values mean that the movement is made in the direction required to reach the goal.

When simulating the rotation and strategy group, a similar technique to reduce computation time was used as when the 8 goals reaching task were simulated. The parameters for each goal and their 45 degrees rotations are computed in advance after the initial basal ganglia training by simulating additional trials. Then, the output of the cerebellum is added to the stored values. Changes in the motor goal are then simulated by recalling a different value from memory. Simulations with the only rotation group are made by solving the complete network.

All simulations were implemented using the neural simulator ANNarchy: a software tool designed for distributed rate-coded or spiking neural networks [109]. The code was written using ANNarchy’s python interface, however, the simulator generated parallel C++ code. Each simulation was ran using 2 threads on a computing server with two AMD EPYC 7352 24-Core processors and 256 GB memory. Each simulation of the whole model takes around 12 hours. We ran 25 simulation in parallel on the same machine.

## Supporting information

S1 Fig Effect of learning speed and noise levels in the performance of the reservoir. We ran multiple simulations with different values for the perturbation frequency (f), the perturbation amplitude (A) and the learning rate (eta). The color in each plot represents the distance between the achieved position and the goal position. Goals were selected randomly but always with an distance of at least 0.5 from the initial hand position. The plots show that low amplitude impede learning as the hand has stayed close to the initial position. With high enough amplitude to produce a strong movement, the network is sensitive to the value of the other parameters. High errors points are intermixed with low error points. High error points are more common when the three parameter values are high.
(EPS)

S2 Fig Activity of the basal ganglia during an example trial. Activation of a goal position in the pre-motor cortex will activate the basal ganglia loop which will select one between the 120 available concrete actions. Each line in the figure correspond to one action channel. In this example the red action is selected. Selection starts by an activation of Striatum D1 cells which then inhibit the SNr. The constant inhibition that reaches the thalamus is then reduced allowing it to activate. Due to its thalamic inputs the motor cortex activates. Finally feedback connection to the striatum further enhance the selection.
(EPS)

S3 Fig Error signal in the cerebellum during the visuomotor adaptation task. Each plot of the figure shows the error signal guiding learning in the model’s cerebellum during the adaptation task under one of the three different conditions. Aiming error is the distance between the current motor goal and the achieved position. On the first two conditions, once a perturbation is introduced the error increases and is then reduced with learning. Removing the perturbation produces a second increase in the error which is again slowly reduced trial by trial. In the STRATEGY condition, the change in the concrete action by the basal ganglia keeps a low error in the cerebellum and avoids learning.
(EPS)

S4 Fig Visuomotor adaptation without the cerebellum. We ran 50 simulations of the rotation + strategy condition where after the initial training with two random goals the cerebellum’s corrections were removed. Once the perturbation is introduced, the model makes a large error which is then reduced after it is instructed to counter the perturbation (trial 103). Different to the previous simulations with the full model, the error stays flat until the model is instructed again. By the end of the simulation no aftereffect is observed. Shadow area next to the curve shows the standard deviation. The variability between simulations is explained by the fact that each time we use a different set of random concrete actions.
(EPS)
