# Correction: The contribution of the basal ganglia and cerebellum to motor learning: A neuro-computational approach

**DOI:** 10.1371/journal.pcbi.1011243

**Published:** 2023-06-22

**Authors:** Javier Baladron, Julien Vitay, Torsten Fietzek, Fred H. Hamker

The following information is missing from the Funding statement: Parts of publication costs were covered by the Deutsche Forschungsgemeinschaft (DFG, German Research Foundation) project number 491193532 and the Chemnitz University of Technology.

The full statement reads as follows:

This study was funded by German Research Foundation (DFG, 416228727)—SFB 1410 Hybrid Societies awarded to F.H. Parts of the salary for Javier Baladron and Torsten Fietzek were covered by the fund above. Parts of publication costs were covered by the Deutsche Forschungsgemeinschaft (DFG, German Research Foundation) project number 491193532 and the Chemnitz University of Technology. The funders had no role in study design, data collection and analysis, decision to publish, or preparation of the manuscript.
